# Behavioral Risk Factor and Primary Healthcare Utilization in South Africa

**DOI:** 10.3390/healthcare10112186

**Published:** 2022-10-31

**Authors:** Ebenezer Toyin Megbowon, Oladipo Olalekan David, Jabulile Lindiwe Makhalima

**Affiliations:** School of Economic Science, North West University, Vanderbijlpark 1900, South Africa

**Keywords:** healthcare utilization, public sector, behavioral risk factor, South Africa

## Abstract

(1) Background: An effective and efficient primary healthcare service is one of the reforms designed to achieve universal healthcare coverage. The success of the reform however depends on the ability to identify factors that could undermine through avoidable use, the effectiveness of various deployed scarce resources. The prevalence of unhealthy lifestyle risk factors that have been identified as a critical public health issue, which stimulate vulnerability and mortality through the development of non-communicable diseases, also have implications for government health spending through healthcare utilization. (2) Objective: This study aims to investigate the effect of behavioral risk factors on primary healthcare utilization in South Africa. (3) Methods: Using the NIDS wave 4 data set and a binary logistic estimation technique, the study is premised on a modified Anderson model of health service utilization. (4) Results: The binary logistic regression estimation results clearly show the intercepting effect of smoking in public primary healthcare utilization. Equally, the effect of these lifestyle behavior risk factors on public PHC is evident in urban communities. (5) Conclusion: This study suggests that there is a need to intensify awareness on the health effect of smoking; strengthen and broaden law that bans smoking; and introduce the screening of smoking patients for recurring counselling sessions and intervention at primary healthcare facilities in the country’s urban communities.

## 1. Introduction

Healthcare service utilization (HSU) is defined as the use of healthcare related services for health promotion and general welfare, prevention of illness, ailment diagnosis, and health status information [1,2]. HSU has a direct link with the healthcare system of a nation and the type of healthcare services that are carried out. HSU and its form can be mirrored from the healthcare-seeking behavior of individuals. Thus, healthcare services are primarily planned and provided based on evidence concerning the pattern of healthcare seeking behaviors and its various defining factors (such as need as well as physical, social, cultural, economic, religious, and demographic factors) [3]. Globally, healthcare service is provided by both the public (that is, the government through the national healthcare system, which is relatively free) and the private sector (provided through profit and non-profit organizations and individuals and is funded by individuals’ contributions) [4]; however, the extent of involvement or size of contribution differs across countries. For instance, in the United State of America, approximately 69.1 percent of healthcare service facilities and delivery are owned and operated by the private sector [5,6]. Unlike the USA, private sector healthcare service provision is predominant with more than 60 percent involvement in some countries (Belgium, Norway, Germany, Switzerland, France, Netherland) but is of lesser importance in others (Denmark, Ireland, Sweden, Norway, UK) [6,7]. In Asia, while more than half of healthcare services are provided by the private sector in Cambodia, China, India, Philippines, Thailand, and Myanmar, it provides a minimal amount of services in other parts of Asia (Fiji, Timor-Leste, New Guinea, and Solomon Islands) [8]. In African health systems as a whole, the private sector accounts for a large proportion of healthcare service delivery [9], nevertheless certain variation exists between countries. While the private sector provides more than 50 percent of healthcare services in Nigeria, in Ghana, South Africa, and Ethiopia a majority (more than 50 percent) of healthcare facilities are owned and operated by the public sector [7,10,11].

Similar to other sectors in an economy, motive, financing strategy and efficiency differentiate public healthcare service from that of the private. The public health service is premised on providing social justice and equality while the private health service has little or no concern for equality in society [4]. For example, [12] noted the difference in pressure faced by both public and private healthcare providers, in that while political and administrative pressures are vital for public healthcare service providers, private providers are faced with competitive pressure that help to optimize their performance. The authors further noted that unlike private healthcare providers, public providers can remain in business despite performing below the ideal level.

Healthcare services are provided at three tiers, that is, primary, secondary, and tertiary tiers. The primary tier is the foremost level of interaction with the health system for individuals irrespective of their health needs. The secondary tier is the second level of an individual’s interaction with a nation’s health system. This tier provides healthcare services that are referred from the primary tier, services at this level are provided by specialists who have more specialized knowledge and skills, usually at district hospitals or private clinics. The tertiary tier services encompass highly specialized healthcare and inpatient care services. Patients referred to this tier need advanced, complex procedures and treatments, which can only be performed by specialists in tertiary hospitals or specific medical institutions [13]. Of all these three tiers, the primary has been put forward to be the most vital for promoting the health of the population of a country. According to [14], “Primary Health Care (PHC) is a whole-of-society approach to health that aims at ensuring the highest possible level of health and well-being and their equitable distribution by focusing on people’s needs and as early as possible along the continuum from health promotion and disease prevention to treatment, rehabilitation and palliative care, and as close as feasible to people’s everyday environment.” The pursuit of the sustainable development goals (SDGs) and a universal health coverage (UHC) resonates and strengthens the idea of an improved primary health care system in the 21st century. PHC entails three inter-related and synergistic components that include comprehensive integrated health services that embrace primary care as well as public health goods and functions as central pieces; multi-sectoral policies and actions to address the upstream and wider determinants of health; and engaging and empowering individuals, families, and communities for increased social participation and enhanced self-care and self-reliance in health [14,15]. PHC is rooted in a commitment to social justice, equity, solidarity, and participation. It is based on the recognition that the enjoyment of the highest attainable standard of health is one of the fundamental rights of every human being without distinction [15]. The extent of success of PHC lies with the extent of involvement of the public sector. However, the realities of socioeconomic disparities, increasing cost of private healthcare procurement, the pursuit of social justice and a reduction in inequality and its effects in several developing countries indicates the need for the public sector (a pro-people sector) to be largely involved in PHC in these countries.

In South Africa, the health of South Africans has been prioritized by the country’s various post-apartheid governments. The entrenched constitutional right to health and an understanding of the imperativeness of human capital health on economic productivity and development justifies the priority of the government on the health sector. South Africa’s health system is dichotomous in nature, however unlike India where the private healthcare service is more utilized than the public healthcare service, the public sector services approximately 80 percent of the population and the private sector takes care of the remaining 20 percent. Public sector healthcare expenditure over the years has been consistently increasing in South Africa. One of the major reasons is that a majority of people utilize public healthcare services due to a lack of access to private medical cover. This has caused a consistent increase in public sector health expenditure. However, despite increased government expenditure, the health situation in the country can only be compared to that of developing countries because the situation reflects an inefficiency in the use of healthcare resources [16,17]. Currently, South Africa faces a disease burden of tuberculosis (TB), a very high prevalence of Human Immunodeficiency Virus (HIV) and Acquired Immune Deficiency Syndrome (AIDS), child and maternal morbidity and mortality, as well as an explosion of life-style related non-communicable diseases, injuries, and violence [18,19,20,21]. The treatment of these diseases largely falls under the primary healthcare tier of the country, meaning that a larger proportion of public funding is needed at this community level of healthcare. It needs to be mentioned that several South African governments since the establishment of a fully democratic government in 1994 have committed to improving PHC; this has been done by enacting policies that widen healthcare services, improve quality of service, promote access, and provide a better health infrastructure [18,22]. However, poor or slow economic growth leading to limited available resources warrants the need and pursuit of cost-effective strategies and intervention in public resources use.

Recently, attention has been on the effect of behavioral risk factors (i.e., unhealthy behaviors, such as smoking, alcohol consumption and physical inactivity) which have been identified as a factor that is increasing the burden of healthcare systems across the world and a challenge to sustainable development goal achievement. Additionally, ref. [23] pointed-out that unhealthy behavior is an amplifying factor in the noticeable growing sickness and death rates, which also could bring about higher HSU, while [24] noted that understanding the effect of unhealthy behaviors warrants examining the dynamic pattern of these behavioral risk factors. This dynamic pattern can be viewed from the perspective of co-occurrence or clustering and transitioning rather than an isolated independent perspective. Understanding the health-seeking pattern or behaviors and its determinants can mostly help government, policy makers, stakeholders, and healthcare service providers in least developed and developing countries to identify relevant intervention and adequately allocate and manage existing resources [3], specifically in public primary healthcare delivery. The objective of this study is to address two questions: (i) which lifestyle risk factor pattern affects primary health care utilization and (ii) does the impact of these lifestyle risk factor patterns of primary healthcare utilization differ across urban and rural areas? The remainder of the study is structured as follows: Section 2 focuses on the literature review, methodological issues are presented in Section 3; Section 4 is the results and the discussion of findings; and Section 5 concludes the study with policy implications.

## 2. Literature Review

Several theories have been put forward and discussed in the literature in relation to healthcare service utilization determinants or demand for healthcare service. Two of these theories are the neoclassical theory of rational consumer behavior and Anderson’s model for healthcare service utilization. The neoclassical (also known as orthodox) economic theory of rational consumer and constrained utility maximization is a major foundation of modern health care demand analysis [25]. The theory focuses on estimating the effects of price and income on utilization of medical services and healthcare expenditure [26]. It assumes that people drive utility directly from the health obtained from the consumption of goods and services, which in this case is medical services, and that individuals choose an outcome that maximizes the utility gained from a particular choice, subject to a health production function and a budget or resource constraint that incorporates income and time [25,26,27]. This derived demand for health care and healthcare service with regard to the effect of access prices of health care is popular in developing countries because the policies of developing countries concentrate on how to improve the population’s access to health care services [26]. In addition, the healthcare service utilization model developed by [28], suggests that healthcare utilization is determined by three key factors, which are predisposing, enabling and need factors at both the individual and contextual level [29]. Predisposing factors can be social demographic characteristics, such as age, race, sex, status which increase one’s need for health services. For instance, an individual who believes a health service is an effective treatment for an ailment is more likely to seek care. Enabling factors facilitate or impede use of health service. Examples of enabling factors could be family support, access to health insurance, one’s community, etc. Need for care represents both self-perceived and actual need for a health service [30]. In all, healthcare service utilization depends not only on income and price but also on individual and household specific variables, such as education, age, and location, among others.

Empirically, evidence from the literature shows that healthcare service utilization is dynamic and that there are diverse factors that influence healthcare service utilization. In line with the central focus of this study, studies that reported unhealthy behavior as one of the independent variables are reported. In terms of smoking, studies of [31,32,33,34,35,36] demonstrated that smoking has a significant effect on primary healthcare, outpatient and inpatient health care service utilization, duration of stay, calls for an ambulance, primary care, and specialist services. No significant relation between the use of tobacco by smokers and former smokers and the number of visits to primary care clinic or tertiary teaching hospital were reported by [37] in the last 12 months in Saudi Arabia. In a similar finding, in a study conducted among older adults in the Irbid Governorate of Jordan, tobacco use was not a significant predictor of PHC services utilization in the past 1, 6, and 12 months [38]. Regarding alcohol, evidence from the empirical literature differs. While some [39,40] showed decreased odds of seeking medical care (HIV test, seeking care, inpatient or outpatient healthcare use) for current alcohol drinkers, other studies [41,42] demonstrated an increased admission rate for moderate and heavy drinkers, and mental health service use. In another study, [43] found that never smoking and non-sedentary behavior were associated with a lower risk of hospitalization, visits to the primary care physician and visits to the medical specialist. Studies [44,45] have also validated the reducing effect of physical activity on healthcare utilization. However, [46,47,48] noted that the association of physical and healthcare utilization is dependent on age. Similarly, regarding the co-occurrence or clustering of behavior, [35] found that participants having seven unhealthy lifestyles are 24 percent less likely to visit a GP for medical consultation. Furthermore, [24] also showed that the odds of attending a past-year primary care visit were approximately 25–35 percent lower for those who engaged in multiple risk behaviors compared to persons engaged in single risk behavior.

Prior studies [49,50,51,52] on healthcare utilization conducted in South Africa seem to not have considered the effect of health risk behavior in their estimation, such bias or the omission of such variable could present an incomplete identification of factors affecting health care utilization. Issues related to public healthcare choice and utilization were examined by [49,50,51,52], without focusing on primary healthcare and the effect of unhealthy behavior; [52] examined determinants of private healthcare choice; and others have looked at healthcare utilization in general, differentiating between public or private, and not accounting for the influence of unhealthy behavior in their estimations. Apparently, research on health risk behaviors and especially primary care utilization has focused on individual health risk behaviors.

## 3. Materials and Methods

### 3.1. Setting, Study Design and Participants

This study was conducted on South Africa. South Africa is one of the countries in the Southern block of Africa. The country has nine provinces (Eastern Cape, Free State, Gauteng, KwaZulu-Natal, Limpopo, Mpumalanga, Northern Cape, North West and Western Cape), a land mass of 1,220,813 km^2^ and shares borders with Namibia, Botswana and Zimbabwe in the North and with Swaziland and Mozambique in the northeast. South Africa is estimated to have a population of approximately 60.14 million people, during the mid-2021 [53]. The study adopted a cross-sectional research design in that the study examines the population at a specific period, which in this case is the year 2017. Furthermore, the research method for this study is a quantitative research method. In terms of selection criteria, the participants examined in this study are individuals aged 18 years and above that can be drawn from the entire dataset. In South Africa, an individual is considered an adult when he or she achieves the age of 18.

### 3.2. Outcome Variable

Primary healthcare utilization is a binary variable, reflecting having had health consultation at a primary healthcare facility or otherwise among adults who reported visiting a public primary healthcare clinic when ill. The question in the NIDS related to the dependent variable is where did the last consultation take place when the respondent last consulted someone about their health? The variable was recoded to a binary variable with assigned values of “1” if the consultation took place at a public primary health clinic (PPHC) and “0” if otherwise.

### 3.3. Explanatory Variables

The main explanatory variable is behavioral risk factors and its clustering. The effect of this variable has been discussed in detail in the literature review section. To capture the pattern of clustering of unhealthy behaviors exhibited by an individual, a STATA command was used to generate an unhealthy behavior variable that has categories of clustering patterns, which reflects unhealthy behavior that is true of an individual at any time. This approach was used to capture clustering dynamics rather than independent isolation of unhealthy behaviors.

Other independent variables are included based on past literature suggesting that they could moderate the effect of lifestyle risk factors on healthcare service utilization. These variables are gender, age, marital status, educational attainment, employment status, and geographical location. The definition of these variables is presented in Table 1.

Gender: Empirical evidence shows that there is an existence of gender differences in healthcare service utilization and health outcomes. Studies have shown that women are more likely to seek medical attention due to their reproductive and biological needs [54] and their perception and consciousness about health, which differs from men [55]. In this study, gender is represented as “1” for male and “0” otherwise. In line with past literature, gender is posited to have a negative a priori expectation.

Frailty and multiple chronic diseases and clinical conditions are higher among older person compared to younger adults [56], thus, it is postulated that older people are more likely to seek medical care than the younger because of their biological needs because of aging. Age in this study is a categorical variable.

Employment status explains individuals’ economic engagement. People who are employed may be more likely to seek medical care at private rather than public primary healthcare facilities because they have medical aids or have the financial resources to meet the cost of private healthcare seeking. In this study, employment status is a dummy variable where “1” is employed and “0” is otherwise. It is expected that employment status will have a negative effect on public primary healthcare service utilization. 

Educational attainment is an indicator reflecting the social and economic status of persons. Individuals with a higher level of education may be more likely to seek health services [57], especially preventive health services, than those with a lower level of education because their knowledge of health and their resources enable them to treat health problems timely. The educational attainment in this study is in three categories and it is expected that their respective effect on public primary healthcare utilization will differ. 

Geography, in terms of location, distance and characteristics of environment, is one of the factors that influences health service use [58]. For instance, the characteristics of urban setting in terms of environmental quality and extent of economic engagement of individuals in urban areas will determine the use of healthcare facilities. Thus, it is posited in this study that the effect of geography on primary healthcare use will either be negative or positive. For the analysis in the study, “1” represents urban, while “0” is otherwise.

Income and wealth are keys to accessing health systems by enabling people to afford health insurance or to pay their cost-sharing requirements to utilize health services. As an indicator of wealth, social and economic status, and financial capacity, those with higher income will likely visit private hospitals and specialists where there is convenience and experience of private care, whereas those with a lower income would only visit publicly owned clinics where no payment is required or cost is minimal. This study therefore suggests that with higher income, public healthcare service utilization will be lowered.

### 3.4. Data Sources and Study Size

This study utilized the survey from the National Income Dynamics Study (NIDS). NIDS is a face-to-face longitudinal survey of individuals and households living in South Africa. It is the first national household panel study in South Africa, which is an initiative of the Department of Planning, Monitoring and Evaluation (DPME) but implemented by the Southern Africa Labour and Development Research Unit (SALDRU) based at the University of Cape Town’s School of Economics [59]. NIDS has been conducted biennially in South Africa since 2008. Specifically, the study utilized wave 4 survey data from the National Income Dynamics Study (NIDS) that was carried out in the year 2015, having 22,737 individuals. A detailed description of the survey’s sampling procedure is available at www.nids.uct.ac.za (accessed on 20 August 2021).

### 3.5. Statistical Methods

Descriptive statistics, such as mean, standard deviation, frequency, percentages, and tables, were used in describing the socio-economic and the demographic to also profile patterns of unhealthy behavior (clustering) and primary healthcare utilization. A binary logistic regression was applied on data obtained for this study to investigate the effect of unhealthy behavior on primary healthcare utilization. The study utilized a binary logistic regression because of the dichotomous nature of the dependent variable. While logit and probit models appear to be similar in that they both produce almost the same result, they differ in the specification of the distribution of error terms and distribution function in that while logistic regression model has a logistic distribution function, probit regression model has a cumulative distribution function. The probability that an individual will utilize a public primary healthcare facility is assumed to be determined by an underlying response variable that captures the actual status of individuals. In the case of public primary healthcare utilization, the underlying response variable following [60] PPHC* is defined by the regression equation

(1) $$PPHC_{i}^{*}=\sum x_{i}\beta +\mu_{i}$$

where $x_{i}\;$represents a vector of the explanatory variables, β is vector of parameters of explanatory variables to be estimated, and $\mu_{i}$ represents the random error term. In Equation (1), $PPHC_{i}^{*}$ is not observable, as it is a latent variable. What is observable is an event represented by a dummy variable *PPHC* defined by;

(2) $$PPPHC_{i}=\left\{\begin{array}{c} 1\;if\;PPHC^{*}>0\\ 0\;if\;Otherwise \end{array}\right.$$

The empirical implicit model function for this study is specified as:

(3) $$PPHC=f\left\{\begin{array}{c} pattern\;of\;clustered\;unhealthy\;behaviour,\;male,\;age,\;marital\;status,\;\\ education\;attainment,\;employment\;status,\;urban,\;income \end{array}\right\}$$

As seen in the specified multivariate logistic regression model in Equation (3), the explanatory variables were included simultaneously in order to obtain the adjusted odds ratios (ORs) that were reported in this study. Adjusted odds ratios are odds ratios that have been adjusted to account for other predictor variables in a model. 

## 4. Results

Table 2 shows that the mean age of respondents in the sample is 36–37 years across total, urban, and rural areas. Going by the distribution of gender across geographical location, Table 2 shows that in both urban and rural areas, the majority (56.82 percent and 60.13 percent) of respondents are female. Furthermore, Table 2 shows the distribution of respondents according to marital status of household heads and indicates that the proportion of individuals that are married ranges between 21 percent and 24 percent across the sampled population in the pool in urban and rural areas, respectively. The results of individual’s educational status, as shown in Table 3, reveal that a larger proportion (57 percent, 58.9 percent and 54.9 percent) attained a post-secondary education. In terms of employment, a larger proportion of those employed are found in urban areas. This is not unexpected because economic activities in South Africa are primarily prevalent in urban areas.

The distribution pattern of unhealthy behaviors among individuals by total and geographical location is presented in Table 3. The table shows that of the 22,720 individuals in the sample, a large proportion of people in both urban and rural areas do not engage in physical exercise. The descriptive statistics carried out shows that the prevalence of clustered unhealthy behavior is higher for urban (33.12 percent) compared to rural (19.24 percent). Clustering of alcohol consumption and physical inactivity (11.62 percent for urban and 7.66 percent for rural), and smoking, alcohol and physical inactivity (10.30 percent for urban and 6.16 percent for rural) are the largest proportion of clustered unhealthy behaviors. This shows that a majority of individuals with more than two unhealthy behaviors are prevalent in the urban areas. Table 4 presents the distribution of primary healthcare utilization by behavioral risk factor in the study sample. The table shows that a majority (53.84 percent) of those that do not engage in physical exercise visited a primary healthcare facility within the last 12 months. A similar pattern is observed for both rural and urban, although the proportion is higher for rural dwellers. 

The multivariable logistic regression result of the effect of lifestyle risk factor clustering on primary healthcare utilization is presented in Table 5. The validity of the estimated result in Table 5 is supported by the goodness-of-fit Pearson χ2 test diagnostic test conducted. The goodness-of-fit test helps to decide if a model fits reasonably well and can be accepted or otherwise. A large *p*-value (that is not significant) indicates that the model is well fitted. In this study, the goodness of fit of the estimated model under the pool context is large, that is, it is not statistically significant as required. Additionally, to further ascertain that the estimated model is unbiased and consistent, a multicollinearity test among the explanatory variables was carried out using the variance inflation factor (VIF). The rule of thumb for this test is that the value must not exceed 10. Thus, based on the vif test value of 1.73 obtained for this study, it is satisfying to conclude that there is no multicollinearity among the variables under consideration. Table 5 shows smoking only was significantly associated with public primary healthcare utilization (AOR = 1.266, 95% CI = 1.006–1.593), smoking and physical inactivity (AOR = 1.335, 95% CI = 1.13–1.577), smoking and alcohol (AOR = 1.324, 95% CI = 1.115–1.571), and smoking, alcohol, and physical inactivity (AOR = 0.142, 95% CI = 0.999–1.305). Likewise, for the pool model, considering other cofounders, the odds of using a public primary healthcare facility is lower for gender (male) (AOR = 0.428, 95% CI = 0.398–0.460), being employed (AOR = 0.858, 95% CI = 0.800–0.920), married (AOR = 0.880, 95% CI = 0.813–0.952), residing in an urban area (AOR = 0.816, 95% CI = 0.765–0.869) and for all educational levels considered. Contrarily, the odds of using a public primary healthcare facility increases with age.

The sensitivity analysis was conducted by disaggregating the respondent by their geographical location (urban and rural). Consequently, a multivariate logistic regression model was estimated for each of the geographical locations. Table 6 clearly shows that the odds of public primary healthcare service utilization is significant and higher with respect to alcohol and physical inactivity (AOR = 1.184, 95% CI = 1.001–1.399), smoking only (AOR = 1.328, 95% CI = 1.004–1.757), smoking and physical inactivity (AOR = 1.439; 95% CI = 1.164–1.778), smoking and alcohol (AOR = 1.416, 95% CI = 1.135–1.767), and smoking, alcohol and physical inactivity (AOR = 1.291, 95% CI = 1.081–1.541). The validity of the urban and rural estimations is revealed by the large and insignificant values of the reported Pearson chi^2^.

## 5. Discussion

This study extends issues and studies related to healthcare utilization in South Africa with a focus on the effect of unhealthy behavior on public primary healthcare, which is the first point of access to the country’s health system and the second largest recipient of government health expenditure in the country. The findings from this study show that not all unhealthy behaviors and their related clustering have a significant impact on public primary healthcare service utilization. For instance, physical inactivity only and alcohol only and the clustering of physical inactivity and alcohol behaviors have lower odds but insignificant value indicating that they do not influence the use of the primary healthcare service. However, smoking only and clustered unhealthy behaviors having smoking, positively and significantly affect the use of the public primary healthcare service. It is clearly seen that smoking is an intercepting unhealthy behavior that if an individual engages in alongside physical inactivity or alcohol consumption will stimulate the use of a public primary healthcare clinic. This finding is consistent with [31], which demonstrated that current smokers visit primary healthcare professional more than nonsmokers. This finding is inconsistent with the finding that adults who engaged in more than one health risk behavior (current smoking plus at-risk drinking) had lower odds of attending primary healthcare check-ups.

The result of gender variable (male) shows a lower and significant odds at *p* < 0.01. The estimated value suggests that being a male reduces the probability of using public primary healthcare. This finding is consistent with [31,61,62,63] for Finland, who all found that men are less likely to seek medical help compared to women. This is however not unexpected because females are known to be more interested in their health status than men; moreover, their need for healthcare utilization is driven by their children’s health as well as productive and maternal health needs, e.g., antenatal, post-natal, and birth-control. Thus, ref. [60] rightly put it that females have more health needs and health awareness than men. The age of individuals having an AOR that is more than one indicates an increasing odds of use of public primary healthcare utilization with an increase in age in the study area. However, the result show the probability of primary healthcare use differs between age categories in that the probability of use is higher for elders (2.039) than for adults (AOR = 1.632). This finding is consistent with [64,65] for India. The result of the regression also reveals lowered odds but statistically significant relationship between employment status and public primary healthcare utilization. The AOR value for the being employed variable suggests that being economically engaged reduces the odds of using a public primary health facility. This is not unexpected because some employed persons have access to medical insurance that could be used for private healthcare, while others when compared to unemployed persons have sufficient financial resources for private healthcare treatment. Moreover, considering the long waiting time at public primary healthcare facilities, an employed person would prefer to trade their financial resources for the long waiting time at the health facility.

The result of this study also shows that being legally married reduces the odds of using the public primary healthcare service. A related finding is reported by [66], where a married elderly variable is twice more likely to prefer private hospitals than the unmarried. Similarly, [67] found that people who are currently married are more likely to use public hospitals than primary health facilities. The AOR value of the geographical location variable (urban) is less than one, and it is significant. This suggests that compared to rural dwellers, urban residents are less likely to seek medical services at a public primary healthcare clinic. This is consistent with the study conducted in Timor-Leste by [61], which demonstrated that rural respondents are 1.3 times more likely to seek health care in a primary care facility than urban residents. Level of education negatively and significantly influences the probability that an individual will use a public primary healthcare facility. This implies that the likelihood that an individual’s use of a public primary healthcare facility will decrease with increasing or higher educational attainment level. Imperatively, the higher the level of educational attainment, the lower the odds of visiting a public health facility. Education is expected to lead to increased earning potential and better knowledge, and it provides higher levels of welfare (such as food security), leading to better health for an individual. This finding is in line with [62,64,65] who found that those with higher education have a higher relative risk of using private facilities than those with incomplete higher schooling. Furthermore, [67] further buttressed that the lowered healthcare standard offered at a public primary healthcare clinic often warrants the probability of those with a higher level of education not using such facilities. The value of income variable in Table 5 is negative and statistically significant though with very minute effect.

The result of the effect of unhealthy behavior patterns and other socioeconomic variables on primary healthcare utilization across urban and rural areas clearly shows that compared to rural areas, these unhealthy behaviors and their clustering significantly increase the use of public primary healthcare services in urban areas. The result of the intercepting effect of smoking behavior in urban areas is consistent with the pattern in the pool estimation. The prevalence of non-communicable diseases (NCDs) and obesity in urban communities compared to rural communities, as noted by [68], is caused by unhealthy behaviors of urban lifestyles, and it explains why these behaviors stimulate use of public PHC in the country’s urban communities. Considering the variables gender and age across geographical location, a similar effect of both gender and age is observed in both rural and urban areas, though the extent of effect differs slightly in that it is larger for rural area dwellers than urban. The reducing effect of employment status, marital status and education attainment on public primary healthcare use is likewise observed across both rural and urban areas, however it is observed that the extent of effect of educational attainment is larger in the urban area compared to the rural area.

There are a few limitations of this study that are identified and must be admitted. First, other dynamics of the outcome variable, such as frequency of visit and the reason for visit (that is, either for preventive or curative care), could have provided an interesting insight on the use of PPHC. Second, lifestyle risk factors information was measured once. Transitional information could have helped to understand how change in unhealthy behavior relates to PPHC utilization. Third, there are some potential confounding factors, such as distance, incidence and severity of disease, that were not accounted for but could be important factors in the use of PPHC facility.

## 6. Conclusions

This study estimated the effects of a pattern of unhealthy behavior (behavioral risk factors) in terms of clustering or combination on public primary healthcare service utilization among adults in South Africa using descriptive statistics and binary logistic regression estimation techniques. Evidence from the binary logistic estimation techniques shows that the probability of using the public primary healthcare service increases with any unhealthy behavior or clustering that includes smoking. Findings further suggest that the effect of these unhealthy behaviors which are clustered around smoking on public primary healthcare use is higher in urban areas. Meaning that smoking is a particular intercepting unhealthy behavior that could aggravate the use of public primary healthcare. Considering the importance of primary healthcare in South Africa, increasing burden of disease, resource constraints in the country, and imperativeness of primary healthcare success, this study suggests that there is a need to intensify awareness of the health effect of smoking, strengthen and broaden law that bans smoking, and strengthen the provision of smoking-cessation services in the primary-care setting, such as recurring counselling sessions and intervention at primary healthcare facilities in the country. 

## Figures and Tables

**Table 1:** Summary description of variables.

Variable	Description of Variable
Dependent Variable	
Public primary healthcare utilization	1 if consulted a health worker at a public health clinic, 0 otherwise
Independent Variable	
Lifestyle risk factor clustering	1 if physical inactivity only
	2 if alcohol only
	3 if alcohol and physical inactivity
	4 if smoking only
	5 if smoking and physical inactivity
	6 if smoking and alcohol only
	7 if smoking, alcohol and physical inactivity
Male	1 if male, 0 otherwise
Age (years)	1 if 18–342 if 35–603 if 61+
Employment status	1 if employed, 0 otherwise
Married	1 if married, 0 otherwise
Education attainment	1 if primary education 2 if secondary education 3 if postsecondary education.
Geographical location	1 if an urban resident, 0 otherwise
Income	Continuous

**Table 2:** Sociodemographic characteristics of sample.

	Pool		Urban		Rural	
	Frequency	%	Frequency	%	Frequency	%
Gender						
Male	9454	41.58	5072	43.18	4382	39.87
Female	13,283	58.42	6675	56.82	6608	60.13
Age (years)						
18–34	12,294	54.07	6361	54.15	5933	53.99
35–60	7468	32.85	4145	35.29	3323	30.24
61+	2975	13.08	1241	10.56	1734	15.78
Employment Status						
Employed	8.733	38.41	5593	47.61	3140	28.57
Otherwise	14,004	61.59	6154	52.39	7850	71.43
Marital Status						
Married	5128	22.57	2802	23.87	2326	21.18
Otherwise	17,592	77.43	8937	76.13	8655	78.82
Geographical Location						
Urban	11,747	51.66				
Rural	10,990	48.34				
Education Attainment						
No Education	1938	8.52	493	4.20	1445	13.15
Primary	4340	19.09	1944	16.55	2396	21.80
Secondary	12,959	57	6921	58.92	6038	54.94
Post-Secondary	3500	15.39	2389	20.34	1111	10.11
Income	Mean = 7654.12		Mean = 9383.45		Mean = 5805.67	
	SD = 13,949		SD = 18,060		SD =6878.66	

**Table 3:** Distribution of lifestyle risk factors by geographical location.

	Urban (%)	Rural (%)	Pool (%)
None	18.35	17.91	18.13
Physical inactivity only	35.07	57.16	47.30
Alcohol only	7.54	4.57	6.10
Alcohol and physical inactivity	11.62	7.66	9.71
Smoking only	2.92	1.12	2.05
Smoking and physical inactivity	5.05	2.47	3.80
Smoking and alcohol only	6.15	2.95	4.60
Smoking, alcohol and physical inactivity	10.30	6.16	8.30

**Table 4:** Distribution of lifestyle risk factors by geographical location of Utilized Public Primary Healthcare Service (PPHC) facility.

	Urban Located PPHC		Rural Located PPHC		Pool	
	(%)	Prob.	(%)	Prob.	(%)	Prob.
None	15.12	<0.001	15.49	<0.001	15.32	<0.001
Physical inactivity only	41.98		63.34		53.84	
Alcohol only	4.77		3.16		3.87	
Alcohol and physical activity	12.10		7.48		9.53	
Smoking only	2.96		0.90		1.81	
Smoking and physical inactivity	6.97		2.48		4.47	
Smoking and alcohol only	5.06		2.24		3.49	
Smoking, alcohol and physical inactivity	11.05		4.92		7.65	

**Table 5:** Multivariate logistic regression result on effect of lifestyle risk factors on public PHC.

	Adjusted Odds Ratios	St.Err.	*p*-Value	[95% Conf. Interval]	VIF
Physical inactivity only	0.935	0.042	0.131	0.856–1.020	2.06
Alcohol only	1.015	0.082	0.851	0.866–1.190	1.29
Smoking only	1.266 **	0.148	0.044	1.006–1.593	1.11
Alcohol and physical inactivity	1.092	0.068	0.160	0.966–1.234	1.41
Smoking and physical inactivity	1.335 ***	0.114	0.001	1.13–1.577	1.13
Smoking and alcohol only	1.324 ***	0.116	0.001	1.115–1.571	1.25
Smoking, alcohol and physical inactivity	1.142 *	0.078	0.052	0.999–1.305	1.43
35–60 years	1.632 ***	0.065	0.000	1.51–1.764	1.51
61+ years	2.039 ***	0.112	0.000	1.832–2.270	1.65
Male	0.428 ***	0.016	0.000	0.398–0.460	1.28
Employment Status	0.858 ***	0.031	0.000	0.800–0.920	1.25
Married	0.880 ***	0.035	0.001	0.813–0.952	1.25
Urban	0.816 ***	0.026	0.000	0.765–0.869	1.13
Primary	0.901 *	0.054	0.079	0.802–1.012	2.88
Secondary	0.638 ***	0.039	0.000	0.567–0.718	4.56
Post-secondary	0.424 ***	0.032	0.000	0.366–0.492	3.14
Income	1.000 ***	0.000	0.000	1.000–1.000	1.08
Constant	1.05	0.077	0.505	0.909–1.213	
Number of obs.	22,720				
LR Chi^2^ (17)	2402.92				
Prob. > Chi^2^	0.0000				
Log likelihood	−12,694.283				
Pseudo R2	0.0865				
Pearson Chi^2^	20,883.87				
Prob. > Chi^2^	0.2045				
Mean vif	1.73				

*** *p* < 0.01, ** *p* < 0.05, * *p* < 0.10.

**Table 6:** Multivariate logistic regression result on effect of lifestyle risk factors on public PHC: By geographical disaggregation.

	Rural				Urban			
	Adjusted Odds Ratios	St.Err	*p*-Value	[95% Conf Interval]	Adjusted Odds Ratios	St.Err.	*p*-Value	[95% Conf Interval]
Physical inactivity only	0.891 *	0.054	0.057	0.791–1.004	0.966	0.064	0.607	0.848–1.101
Alcohol only	1.026	0.124	0.835	0.808–1.301	1.045	0.115	0.689	0.842–1.297
Alcohol and physical inactivity	0.992	0.092	0.932	0.827–1.190	1.184 **	0.101	0.048	1.001–1.399
Smoking only	1.176	0.254	0.452	0.771–1.795	1.328 **	0.190	0.047	1.004–1.757
Smoking and physical inactivity	1.149	0.166	0.336	0.866–1.525	1.439 ***	0.155	0.001	1.164–1.778
Smoking and alcohol only	1.214	0.173	0.173	0.919–1.604	1.416 ***	0.160	0.002	1.135–1.767
Smoking, alcohol and physical inactivity	0.928	0.100	0.489	0.752–1.146	1.291 ***	0.117	0.005	1.081–1.541
35–60 years	1.644 ***	0.094	0.000	1.47–1.838	1.636 ***	0.091	0.000	1.468–1.823
61+ years	1.917 ***	0.146	0.000	1.652–2.226	2.237 ***	0.178	0.000	1.914–2.615
Male	0.416 ***	0.022	0.000	0.376–0.461	0.446 ***	0.023	0.000	0.403–0.494
Employment status	0.896 **	0.046	0.034	0.810–0.992	0.823 ***	0.041	0.000	0.747–0.906
Married	0.920	0.051	0.131	0.825–1.025	0.856 ***	0.051	0.009	0.763–0.962
Education attainment								
Primary	0.865 **	0.063	0.047	0.750–0.998	0.88	0.094	0.235	0.713–1.086
Secondary	0.632 ***	0.048	0.000	0.544–0.734	0.596 ***	0.063	0.000	0.484–0.734
Post-secondary	0.512 ***	0.052	0.000	0.419–0.626	0.349 ***	0.042	0.000	0.275–0.442
Income	1.000 ***	0.000	0.000	1.000–1.000	1.000 ***	0.000	0.000	1.000–1.000
Constant	1.011	0.096	0.913	0.838–1.218	0.95	0.117	0.680	0.746–1.210
Number of obs.	10,981				11,739			
LR Chi^2^ (17)	949.16				1327.12			
Prob > Chi^2^	0.0000				0.0000			
Log likelihood	−6604.6334				−6051.8766			
Pseudo R2	0.0670				0.0988			
Pearson Chi^2^	9945.18				10,987.25			
Prob > Chi^2^	0.2012				0.2167			

*** *p* < 0.01, ** *p* < 0.05, * *p* < 0.10.

## Data Availability

The datasets used and/or analyzed during the current study are publicly available at DOI: https://doi.org/10.25828/fw3h-v708. However, the cleaned data can be obtained from the corresponding author on reasonable request.
